# Potential effects of genetic variations in fusion protein on the virulence of human respiratory syncytial virus

**DOI:** 10.3389/fimmu.2026.1841974

**Published:** 2026-07-03

**Authors:** Jingjing Song, Na Wang, Zhen Zhu, Naiying Mao, Hai Li, Jinhua Song, Lei Cao, Baicheng Xia, Min Liao, Wuyang Zhu, Yan Zhang

**Affiliations:** 1 National Key Laboratory of Intelligent Tracking and Forecasting for Infectious Disease, National Health Commission (NHC) Key Laboratory of Medical Virology and Viral Diseases, National Institute for Viral Disease Control and Prevention, Chinese Center for Disease Control and Prevention, Beijing, China; 2 Infectious Diseases Translational Research Programme, Department of Microbiology and Immunology, National University of Singapore, Singapore, Singapore; 3 Institute of Synthetic Biology Product Quality and Safety (Secretariat of the Biosafety Coordination Mechanism of SAMR), Chinese Academy of Quality and Inspection & Testing, Beijing, China; 4 Key Laboratory of Animal Virology of Ministry of Agriculture, Zhejiang University, Hangzhou, Zhejiang, China

**Keywords:** fusion protein, human respiratory syncytial virus, pathogenesis, reverse genetics, virulence

## Abstract

**Introduction:**

The fusion glycoprotein (F protein) of human respiratory syncytial virus (HRSV) critically determines viral infectivity and host immune recognition. This study evaluated the effects of F protein genetic variations from the globally dominant HRSV genotypes ON1 and BA9 on viral replication dynamics and pathogenicity.

**Methods:**

Using a reverse genetics system, two recombinant HRSV strains, rLong-BJ1903-AF and rLong-SY2103-BF, were generated by replacing the F gene in the isogenic Long-bacterial artificial chromosome (BAC) backbone with F genes derived from the clinical isolates BJ19-03 (ON1 genotype) and SY21-03 (BA9 genotype), respectively. Their phenotypes were assessed in cellular and animal models.

**Results:**

*In vitro*, both recombinant viruses showed attenuated replication kinetics compared with the parental Long-BAC strain, with peak viral titers delayed by at least 12 h post-infection. *In vivo*, both recombinant viruses caused less severe disease than Long-BAC. Among them, rLong-SY2103-BF displayed greater pathogenicity than rLong-BJ1903-AF, as shown by slower body weight recovery, higher lung viral loads, elevated proinflammatory cytokine levels, and marked pulmonary pathological alterations.

**Discussion:**

These findings indicate that both recombinant viruses exhibit reduced replication capacity and less severe pathogenic phenotypes compared with the Long-BAC parental strain in this F-gene replacement system, providing a foundation for antiviral and immunological strategies targeting HRSV.

## Introduction

The human respiratory syncytial virus (HRSV) is the predominant etiological agent of acute lower respiratory infections (ALRIs) in infants. Global epidemiological data from 2019 estimates indicate ≥ 33 million HRSV-associated ALRIs worldwide, causing approximately 3.4 million hospitalizations and over 100, 000 pediatric fatalities and imposing a significant economic burden on healthcare systems and families (1). Given its significant public health impact, the World Health Organization has prioritized the development of effective vaccines and antiviral treatments to mitigate the burden of HRSV infections.

HRSV is an enveloped, non-segmented, negative-sense, single-stranded RNA virus belonging to the genus *Orthopneumovirus* in the family Pneumoviridae. Its genome spans approximately 15.2 kb in length and encodes 11 proteins essential for viral replication, assembly, and pathogenesis (2). Among these, the fusion glycoprotein (F protein) is critical for viral infectivity and host immune recognition because it mediates membrane fusion to enable entry of the viral ribonucleoprotein complex into the host cytoplasm (3). Structural studies have revealed the pre-fusion (pre-F) conformation as the principal immunodominant form, harboring key antigenic sites that can elicit potent neutralizing antibodies. Thus, pre-F is a primary target for HRSV vaccine and therapeutic development (4). Three pre-F-based recombinant protein vaccines and two monoclonal antibodies have recently been approved for the prevention of severe HRSV-associated ALRIs in high-risk populations (5–8).

Molecular surveillance data have been used to categorize HRSV into two major subtypes, HRSVA and HRSVB, which are further subdivided into 15 and 28 genotypes, respectively (9). Among these, genotypes ON1 (HRSVA) and BA9 (HRSVB) currently dominate global circulation. Recent studies have revealed that both subtypes exhibit high-frequency polymorphisms at key antigenic sites in the F protein (10). Notably, ON1 and BA9 strains harbor multiple amino acid substitutions in key antigenic sites of the F protein, which may affect antigenic properties and potentially contribute to immune escape or reduced sensitivity to certain monoclonal antibodies. Despite the global predominance of these two genotypes, whether such mutations directly enhance viral pathogenicity remains unknown, as host immune status and other confounders complicate clinical correlations (11).

To systematically investigate the functional impact of contemporary F protein mutations, we employed a reverse genetics approach using the prototype Long strain as the backbone. The Long strain was selected for several key reasons: (1) it represents a well-characterized prototype with established reverse genetics tools and complete genomic annotation, facilitating reliable manipulation and recovery of recombinant viruses; (2) it provides a standardized reference point that enables direct comparison of contemporary mutations while controlling for other genomic variables; and (3) previous studies have extensively validated this backbone for reverse genetics applications, ensuring reproducible results and comparability with published literature. This experimental design allows for the precise attribution of phenotypic differences to F protein variations rather than confounding genomic factors.

Based on documented mutations in key functional domains of the HRSV F protein and their potential roles in viral-host interactions, we hypothesized that contemporary F protein variants from the globally dominant ON1 and BA9 genotypes would differentially influence viral replication and pathogenicity compared with the historical Long strain. We further predicted that F protein sequence variations in representative ON1 and BA9 clinical strains may contribute to distinct virulence phenotypes, manifested as differences in replication efficiency, host immune responses, and disease severity.

In this study, we developed a bacterial artificial chromosome (BAC)-based reverse genetics system by replacing the F gene in prototypic HRSV strains with corresponding sequences derived from the representative genotype ON1 and BA9 clinical strains. Using these recombinant HRSVs (rRSVs), we conducted a systematic evaluation of the phenotypic effects associated with F protein variations from ON1 and BA9 clinical strains, focusing on viral replication kinetics, pathogenic profiles, and host immune modulation across cellular and animal models. These investigations will provide insights into genotype-dependent determinants of HRSV pathogenicity and antigenic variability and lay the foundation for optimizing therapeutic and preventive strategies against HRSV-related diseases.

## Materials and methods

### Ethics statement

All animal experiments were performed at the Laboratory Animal Center of the Chinese Center for Disease Control and Prevention (China CDC). Female BALB/c mice (aged 8 weeks) were obtained from Beijing Vital River Laboratory Animal Technology Co., Ltd. This study strictly adhered to the institutional animal ethics guidelines and was approved by the designated review committee of the Chinese Center for Disease Control and Prevention (ethics approval number: 20220525058).

### Cell lines and virus strains

The HEp-2 was preserved at the China CDC, and the BSR T7/9 cell line stably expressing T7 RNA polymerase was provided by Prof. Wuyang Zhu (China CDC). Both cell lines were cultured in Dulbecco’s modified Eagle’s medium (Gibco) supplemented with 10% fetal bovine serum (Gibco) and 1% penicillin-streptomycin, and they were maintained at 37 °C with 5% carbon dioxide (CO_2_). The BSR T7/9 cell culture medium was supplemented with 1 mg/mL G418 (Invitrogen, USA) to maintain selection pressure for the transfected cells. Three HRSV strains were used in this study, namely the prototype strain Long (ATCC VR-26; GenBank accession number: AY911262) and the representative genotype ON1 (BJ19–03 from hospitalized children in Beijing, 2019) and BA9 (SY21–03 from an outbreak at a postpartum care center in Liaoning Province, 2021) clinical strains obtained from the Institute for Viral Disease Control and Prevention, China CDC.

### Plasmid construction and recombinant virus generation

**Figure 1:**
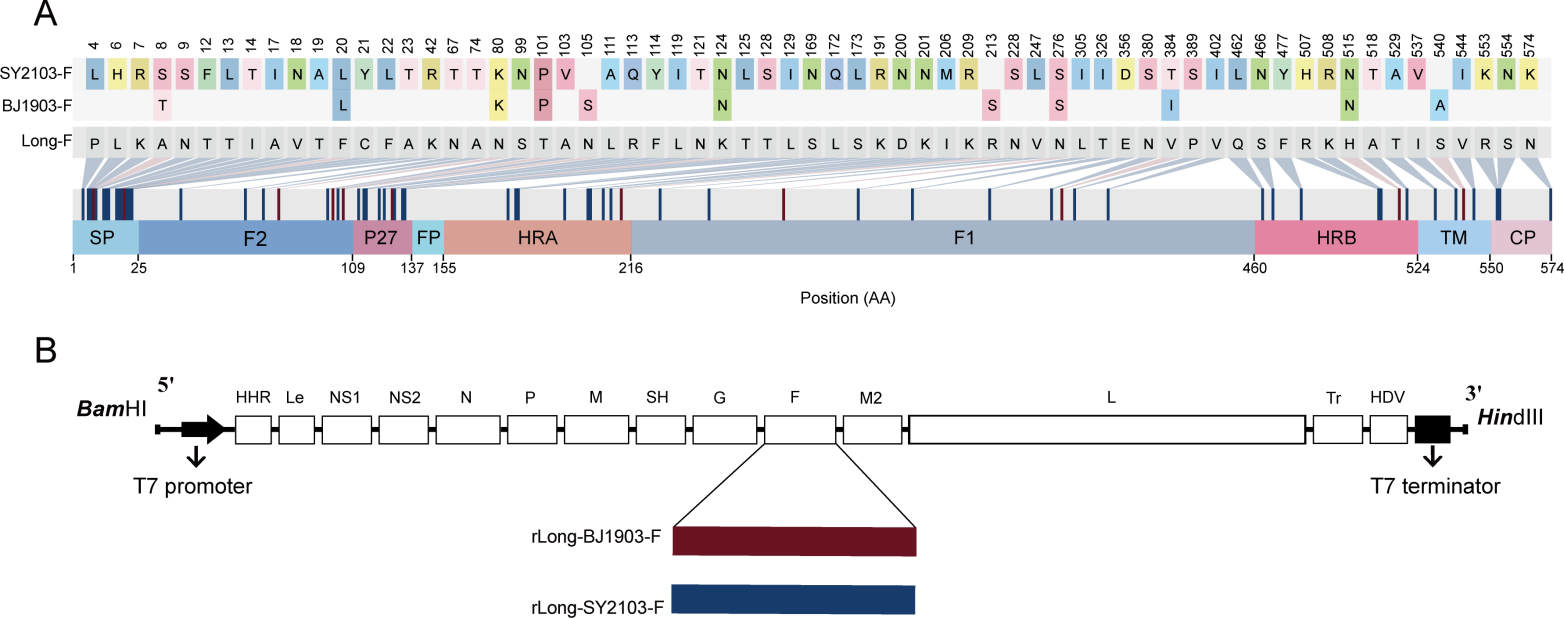
Construction of a full-length HRSV plasmid. **(A)** Amino acid alignment of the F proteins from the prototype Long strain and two representative clinical strains (BJ19–03 and SY21-03). Functional domains of the F protein are annotated below the alignment. These include signal peptide (SP), fusion protein subunit 2 (F2), peptide 27 (P27), fusion peptide (FP), heptad repeat A (HRA), fusion protein subunit 1 (F1), heptad repeat B (HRB), transmembrane domain (TM), and cytoplasmic tail (CP); **(B)** Schematic diagram of the recombinant HRSV strains rLong-BJ1903-AF and rLong-SY2103-BF, generated by inserting the F gene from BJ19–03 and SY21-03, respectively, into the backbone of the prototype Long strain under a T7 promoter. HRSV, human respiratory syncytial virus; HHR, hammerhead ribozyme; Le, leader; NS1/2, nonstructural proteins 1 and 2; N, nucleoprotein; P, phosphoprotein; M, matrix protein; SH, small hydrophobic protein; G, attachment glycoprotein; F, fusion protein; M2, matrix protein 2; L, large polymerase protein; Tr, trailer; HDV, hepatitis delta virus ribozyme.

Based on the BAC vector in HEp-2 cells, the full-length HRSV plasmid was engineered using the genomic sequence of the prototype HRSV strain Long (Long-BAC). Using this plasmid backbone, two recombinant chimeric viruses, namely rLong-BJ1903-AF and rLong-SY2103-BF were generated through targeted replacement of the F gene with those derived from the clinical strains BJ19-03 (genotype ON1) and SY21-03 (genotype BA9), respectively. All plasmids, including the full-length infectious clones and helper plasmids encoding codon-optimized N, P, L, and M2-1 (derived from the Long strain), were synthesized by GenScript (Nanjing, China). Briefly, the full-length genomic cDNA of the HRSV Long strain was cloned into a BAC-based plasmid downstream of the T7 promoter, with a hammerhead ribozyme sequence positioned at the 5′ end and an HDV ribozyme/T7 terminator cassette at the 3′ end, generating the Long-BAC full-length clone (**Figure 1B**). The F open reading frame of the Long strain was subsequently replaced with the corresponding F gene sequences from BJ19–03 or SY21–03 to generate chimeric full-length clones. The integrity of the resulting clones was confirmed by Sanger sequencing.

To assess the helper plasmid functionality, a minigenome (PBR322-RSV-EGFP) (GenScript, Nanjing, China) was constructed using the PBR322 vector, incorporating a T7 promoter, hammerhead ribozyme, genomic segments (leader, trailer, non-coding, and gene start/end sequences) from the HRSV strain Long, an EGFP reporter, hepatitis delta virus ribozyme, and a T7 terminator. Helper plasmid activity was evaluated by co-transfecting PBR322-RSV-EGFP with the four helper plasmids into BSR T7/9 cells and monitoring EGFP expression using a fluorescence microscope.

### Recovery of recombinant viruses

The recombinant plasmids were co-transfected with four helper plasmids into BSR T7/9 cells using Lipofectamine 3000 (Invitrogen, CA, USA) following the manufacturer’s instructions and incubated at 37°C and 5% CO_2_. The viral progeny were serially propagated in HEp-2 cells until a typical cytopathic effect was observed in subsequent experiments. The sequence accuracy and integrity of the recombinant viruses were validated by Sanger sequencing of complete viral genomes amplified by one-step RT-PCR from rescued viruses and P10 HEp-2 passaged viruses, using published primers covering all HRSV genomic regions (Sangon Biotech, Shanghai, China) (12).

### Recombinant virus identification with IFA

The infected HEp-2 cells were fixed in 4% paraformaldehyde (PFA) for 15 min. Non-specific binding sites were blocked with 5% bovine serum albumin (BSA) at 37 °C for 1 h. Cells were incubated with an F protein-specific Palivizumab monoclonal antibody (AstraZeneca, UK). Subsequently, fluorescein isothiocyanate (FITC)-conjugated goat anti-human IgG (H+L) secondary antibody (Beyotime, Shanghai, China) was applied at 37 °C for 45 min under light-protected conditions. Cell nuclei were stained with 4′, 6-diamidino-2-phenylindole (DAPI; Sigma, USA) for 10 min, and fluorescence imaging was performed using a fluorescence microscope (Leica, Germany).

### Viral multistep growth curve analysis

HEp-2 cells were simultaneously infected with recombinant viruses rLong-BJ1903-AF, rLong-SY2103-BF, and the parental control strain Long-BAC at an MOI of 0.1. Following infection, the cells were harvested at predetermined time points (0–90 hpi), and total RNA was extracted using the QIAamp Viral RNA Mini kit (Qiagen, USA). The viral genomic RNA load at each time point was quantified using digital RT-PCR (STILLA, France) with specific primers and probes targeting the conserved N gene region, as previously described (13). The primer and probe sequences were as follows: RSA-F, 5′-AGATCAACTTCTGTCATCCAGCAA-3′; RSA-R, 5′-ATTGATACTCCTAATTATGATGTGC-3′; and RSA probe, 5′-CACCATCCAACGGAGCACAGGAGAT-3′. The 25 μL reaction mixture contained 12.5 μL of (RT-) qPCR ToughMix 2×, 2.5 μL each of forward primer (10 μM), reverse primer (10 μM), fluorescein (1 μM), and RNA template (<1.5 μg), 0.625 μL of probe (10 μM), and 1.875 μL nuclease-free H_2_O. Thermal cycling conditions consisted of reverse transcription at 50 °C for 10 min, initial denaturation at 95 °C for 1 min, followed by 45 cycles of 95 °C for 10 s and 60 °C for 1 min. The viral multiple-step growth curve was plotted using the GraphPad Prism 8.0 software (GraphPad, CA, USA).

### Plaque assay for virus titer determination

The recombinant (rLong-BJ1903-AF and rLong-SY2103-BF) and parental (Long-BAC) strains were serially diluted in 10-fold increments from 10^–1^ to 10^-10^. HEp-2 cells were seeded into 24-well plates at a density of 1 × 10^5^ cells per well and incubated at 37 °C until 80–90% monolayer confluency was achieved. Viral dilutions were inoculated onto the cell monolayers and adsorbed at 37 °C for 1 h to ensure viral binding. Subsequently, the inoculum was aspirated, and the cells were overlaid with methylcellulose overlay minimal essential medium (Solarbio Life Sciences, China). Next, the plates were incubated at 37 °C for 5 days for plaque development. The cells were fixed with pre-cooled 4% PFA and stained with 1% crystal violet. Plaques were quantified, and viral titers were calculated as plaque-forming units per mL (PFU/mL).

### Intranasal infection of BALB/c mice with recombinant viruses

BALB/c mice (8 weeks old; female; n = 6 per group) were anesthetized with inhaled isoflurane in oxygen using 3% for induction and 1.5-2% for maintenance. Under anesthesia, mice were intranasally inoculated with 50 μL of virus suspension per mouse, corresponding to 5 × 10^5^ PFU/mouse for rLong-BJ1903-AF, 1.1 × 10^5^ PFU/mouse for rLong-SY2103-BF, and 5 × 10^5^ PFU/mouse for Long-BAC. The different inoculation doses reflected the available infectious titers of the rescued virus stocks after propagation and were considered when interpreting the *in vivo* pathogenicity comparisons. Mice that received an equivalent volume of cell culture supernatant served as mock-infected controls. Body weight was monitored daily. At designated time points (2, 4, 6, and 8 days post-infection), mice were euthanized by gradual-fill CO_2_ inhalation in a chamber at a displacement rate of 30-70% of the chamber volume per minute, and death was confirmed by cessation of respiration before tissue collection.

### Pulmonary histopathology

After euthanasia was completed and confirmed at 4 and 6 dpi, lung tissues were promptly excised and fixed in 4% PFA, subsequently embedded in paraffin, and sectioned into 3–5 μm slices for histological analysis. Tissue sections were stained with H&E to comprehensively assess pulmonary pathology. Histopathological alterations, including alveolar wall thickening, interstitial pneumonia, bronchiolitis, and alveolitis, were evaluated and scored according to a scoring system ranging from 0 to 4: 0 (normal tissue architecture), 1 (mild alterations), 2 (moderate changes), 3 (significant structural modifications), and 4 (severe pathological damage). The total pulmonary pathology score represents the cumulative score across the evaluated lesion categories.

### Quantitation of lung viral load

Right lung tissues were collected from the mice at 4 and 6 dpi with recombinant viruses. After grinding and centrifugation, the supernatants were carefully collected and used for nucleic acid extraction. Subsequently, the viral load in the lung tissues was accurately quantified using digital RT-PCR.

### Cytokine analysis

The levels of cytokines, including IL-5, IL-10, IL-6, IL-1β, and TNF-α, in serum samples were measured at 4 and 6 dpi using enzyme-linked immunosorbent assay kits (DAKEWE Biotech, China). The entire detection process was performed in strict accordance with the manufacturer’s instructions to ensure result reliability and accuracy.

### Sequence variation analysis and statistical methodology

Amino acid sequence variations between the representative and prototype strains were analyzed through multiple sequence alignment visualization using Snipit (https://github.com/aineniamh/snipit). Statistical analyses were performed using GraphPad Prism (v8.0), with all functional assays containing ≥ three independent biological replicates. Between-group comparisons were performed using two-tailed Student’s t-tests. Multi-group comparisons were performed using a two-way ANOVA followed by Tukey’s multiple comparisons test, as appropriate. Statistical significance was defined as *p* < 0.05 throughout the analyses.

## Results

### Amino acid variations in the F gene of the representative strains used for HRSV full-length plasmid construction

To investigate the impact of F protein mutations from genotypes ON1 and BA9 on HRSV virulence, prototype Long and clinical strains representing the two genotypes (ON1: BJ19-03; BA9: SY21-03) were selected in this study. Comparative analysis revealed 11 and 62 amino acid differences in BJ19–03 and SY21-03, respectively, relative to the prototype Long strain. Notably, 59 amino acid differences were identified between the two clinical strains. These sequence variations were distributed across multiple F protein functional domains (**Figure 1A**). It should be noted that these differences represent sequence variations relative to the Long strain and may include natural polymorphisms, subtype associated divergence, and strain specific differences, rather than genotype specific mutations alone.

Compared with the Long strain, BJ19–03 and SY21–03 exhibited 2 and 15 amino acid differences, respectively, in the signal peptide region; 3 and 8 in the F2 region; 1 and 9 in the P27 region; no amino acid differences were found in the fusion peptide region, which appeared to be the most conserved; 1 and 9 in heptad repeat A (HRA); 2 and 10 in F1; 1 and 7 in HRB; 1 and 3 in the transmembrane domain; 0 and 3 in the cytoplasmic tail (**Figure 1A**).

In addition, several amino acid differences were located within key antigenic sites. Although Sites III, IV, and V remained highly conserved, variations were observed in Sites Ø, I, and II. Notably, Site Ø, a critical target of the monoclonal antibody nirsevimab, contained four amino acid differences in SY21–03 relative to the Long strain (D200N, K201N, I206M, and K209R). Site II, which is targeted by palivizumab, showed a shared difference at residue 276 (N276S) in both strains relative to Long. At Site I, BJ19–03 contained one amino acid difference (V384I), whereas SY21–03 contained three differences (N380S, V384T, and P389S) relative to Long (**Figure 1A**).

### Validation of helper plasmid activity

Four helper plasmids encoding nucleoprotein (N), phosphoprotein (P), large polymerase protein (L), and matrix protein 2-1 (M2-1) were co-transfected with the minigenome plasmid PBR322-RSV-enhanced green fluorescent protein (EGFP) into BSR T7/9 cells. Distinct green fluorescence, observed using a fluorescence microscope at 48 h post-transfection, revealed the functional expression of the helper plasmids and their ability to support minigenome replication and transcription.

### Construction and identification of recombinant viruses

**Figure 2:**
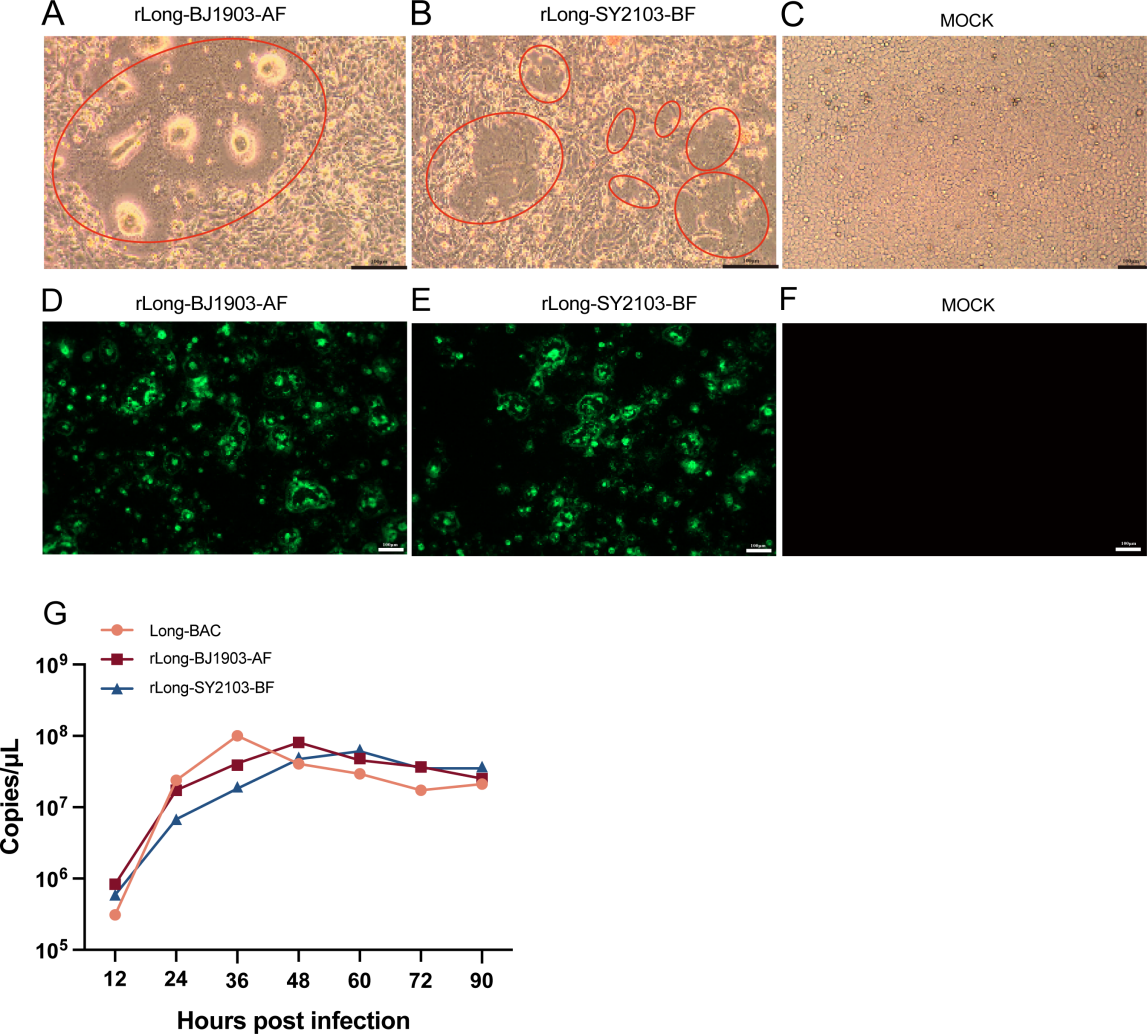
Characterization of recombinant HRSVs (rRSVs). Cellular-level characterization of rRSVs; **(A–C)** Cytopathic effect (CPE) induced by infecting the human laryngeal epidermoid carcinoma cell line (HEp-2) with recombinant viruses rLong-BJ1903-AF and rLong-SY2103-BF, alongside a mock-infected cell control. Syncytial cells were demarcated using red circles; **(D–F)** Immunofluorescence assay detection of viral antigen in infected HEp-2 cells using palivizumab as the primary antibody. Positive fluorescent signals were evident in cells infected with rLong-BJ1903-AF and rLong-SY2103-BF, whereas no signals were observed in mock-infected controls; **(G)** Multistep growth curves of rRSV strains, including the parental Long-BAC backbone and the two recombinants (rLong-BJ1903-AF and rLong-SY2103-BF), in HEp-2 cells. Viral RNA levels were quantified digital RT-PCR.

The F gene in the Long-BAC backbone was replaced with F gene sequences derived from clinical isolates BJ19–03 and SY21-03, generating recombinant infectious clones designated as Long-BJ1903-AF and Long-SY2103-BF, respectively (**Figure 1B**). To facilitate viral recovery, these clones were co-transfected with the four helper plasmids into BSR T7/9 cells. Characteristic syncytium formation was observed (**Figures 2A, B**), followed by serial passaging of the human laryngeal epidermoid carcinoma cell line (HEp-2) to amplify the rescued viruses, which were named rLong-BJ1903-AF and rLong-SY2103-BF.

HEp-2 cell infection with Passage 3 (P3) viral strains caused extensive cell fusion at 48 h post-infection (hpi). Subsequent indirect immunofluorescence assay (IFA) detection using Palivizumab monoclonal antibodies revealed the successful rescue of both recombinant viruses, as evidenced by the distinct green fluorescence in the infected cells (**Figures 2D–F**).

Furthermore, recombinant viruses at passage 3 (P3) and passage 10 (P10) were subjected to whole-genome consensus sequencing using the Sanger method. The sequencing results revealed that the F gene sequences of rLong-BJ1903-AF and rLong-SY2103-BF remained identical to those of the corresponding clinical isolate derived F genes, whereas the sequences of other genomic regions remained identical to those of the prototype Long strain. These findings indicate that both rescued recombinant viruses exhibited stable genetic characteristics at the consensus sequence level during cell culture passage.

### Rescued viruses exhibit distinct replication kinetics *in vitro*

Quantitative plaque assays performed on P10 virus stocks revealed significant titer differences, with rLong-BJ1903-AF achieving 2×10^6^ PFU/mL compared with rLong-SY2103-BF at 5×10^5^ PFU/mL. To characterize replication dynamics, HEp-2cells were infected with viruses at a multiplicity of infection (MOI) of 0.1. Serial quantification of viral genomes via digital reverse transcription-polymerase chain reaction (RT-PCR) at 12-hour intervals (12–90 hpi) revealed similar growth patterns: both strains exhibited exponential replication phases at 12–48 hpi, peaking at 48 (8.0×10^7^ cp/μL; rLong-BJ1903-AF) and 60 (4.7×10^7^ cp/μL; rLong-SY2103-BF) hpi, with peak viral genomic RNA loads delayed by ≥ 12 h compared with that of the parental Long-BAC strain (**Figure 2G**).

### Body weight and lung virus load in mouse model

**Figure 3:**
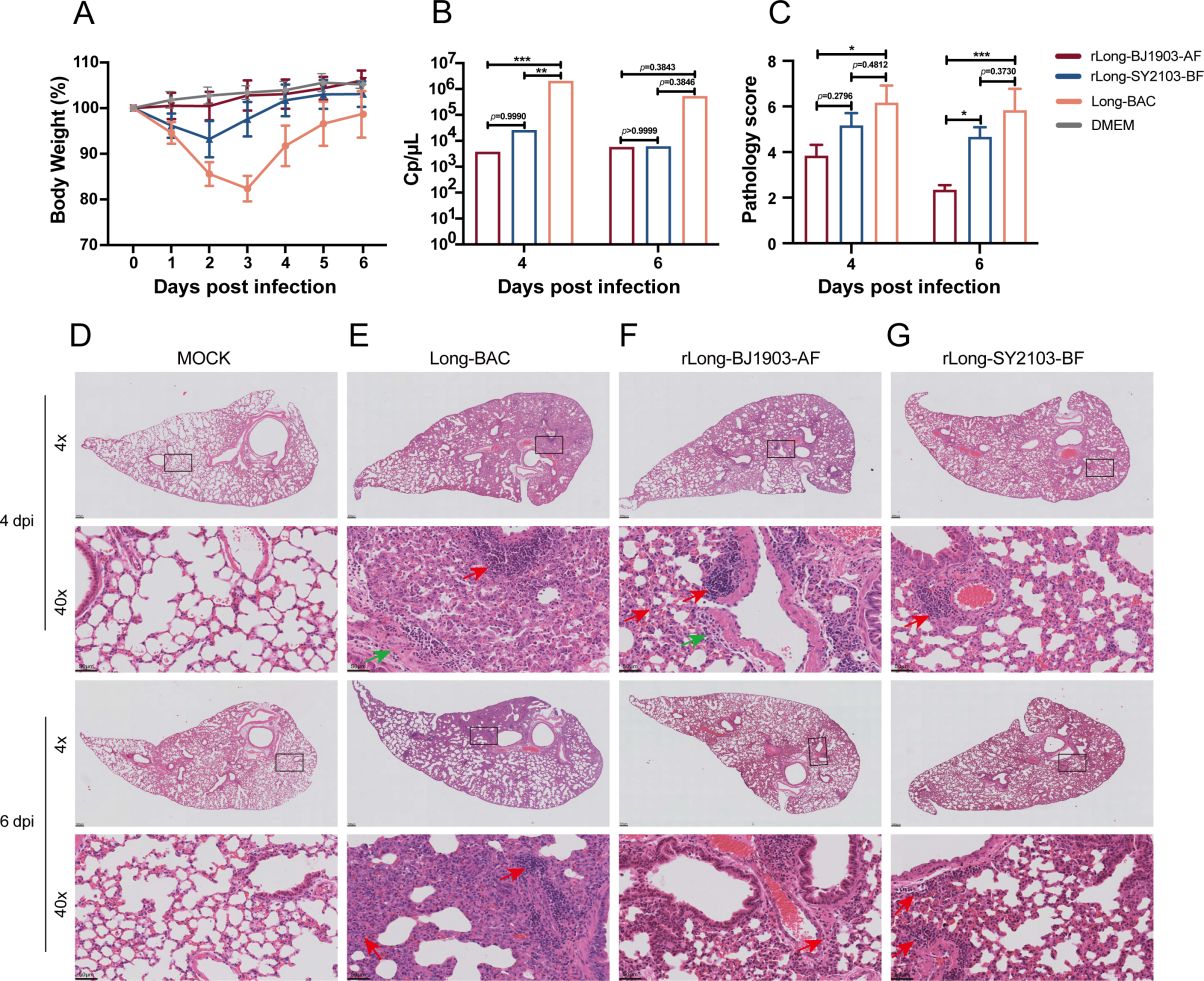
Pathogenicity assessment of rRSVs in BALB/c mice. **(A)** Body weight changes in infected mice over 6 dpi; **(B)** Viral loads in lung homogenates at 4 and 6 dpi, quantified using digital PCR and expressed as viral RNA copies/μL; **(C)** Pulmonary pathology scores derived from histopathological evaluation of hematoxylin and eosin (H&E)-stained lung sections. Statistical analysis was conducted using a two-way ANOVA followed by Tukey’s multiple comparisons test, with significance levels indicated as *p < 0.05, **p < 0.01, and ***p < 0.001. Data are presented as mean ± SD; **(D–G)** Representative histopathological changes in the lungs of BALB/c mice at 4 and 6 dpi for mock-treated controls, Long-BAC, rLong-BJ1903-AF, and rLong-SY2103-BF. Low-magnification (4×) and high-magnification (40×) images are shown to illustrate pathological alterations. Red arrows denote peribronchial and alveolar lymphocytic infiltration, whereas green arrows highlight neutrophil infiltration. Scale bars correspond to 500 (4×) and 50 (40×) μm.

Mice infected with rLong-BJ1903-AF showed no significant weight loss. In contrast, rLong-SY2103-BF infection induced a progressive but modest weight decline, reaching its lowest point at 2 dpi (6.8% decrease), followed by gradual recovery. Mice infected with parental Long-BAC displayed a sustained weight loss trajectory, peaking at 3 dpi (a 17.6% decrease) (**Figure 3A**).

The pulmonary viral loads were measured at 4 and 6 dpi using a digital RT-PCR assay. At 4 dpi, the Long-BAC-infected group exhibited a significantly higher viral load (1.9×10^6^ copies/μL) than the rLong-BJ1903-AF (3.6×10^3^copies/μL, *p* < 0.001) and rLong-SY2103-BF (2.4×10^4^ copies/μL, *p* < 0.01) groups. In contrast, no statistically significant differences in viral load were detected among the three groups at 6 dpi, although the Long-BAC-infected group showed a higher viral load than the other two recombinant viruses (**Figure 3B**).

### Pulmonary pathology analysis

To assess the pathogenic effects of the rescued viruses on murine lungs, lung tissues were collected from BALB/c mice at 4 and 6 dpi and processed for histopathological evaluation using hematoxylin and eosin (H&E) staining (**Figures 3D–G**). At 4 dpi, all infected groups exhibited significant pathological alterations, with the Long-BAC group demonstrating the most severe lesions characterized by alveolar septal fusion, alveolar lumen narrowing, lymphocytic infiltration, scattered neutrophil accumulation, and proteinaceous exudates. The Long-BAC group had a higher pathological score than rLong-BJ1903-AF and rLong-SY2103-BF groups, showing statistically significant differences compared with the rLong-BJ1903-AF group (*p* < 0.05). Notably, at 6 dpi, the Long-BAC group maintained a significantly elevated pathological score relative to the other two groups, with a highly significant difference compared with the rLong-BJ1903-AF group (*p* < 0.001); a statistically significant difference was also observed between the rLong-BJ1903-AF and rLong-SY2103-BF groups (*p* < 0.05) (**Figure 3C**).

### Cytokine expression analysis

**Figure 4:**
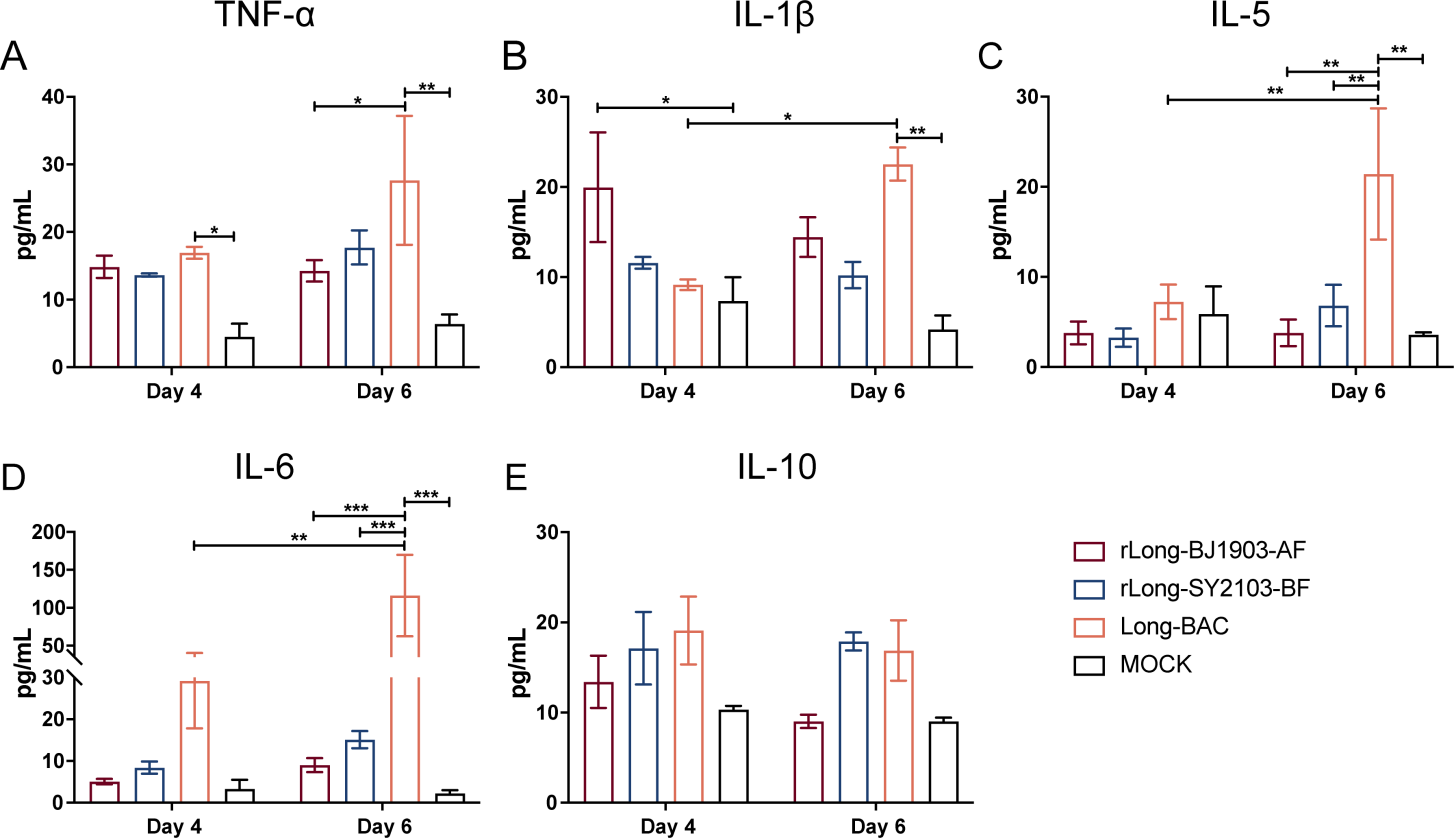
Characterization of serum cytokine concentrations at 4 and 6 dpi. **(A–E)** Statistical analysis was performed using a two-way ANOVA followed by Tukey’s multiple comparisons. Statistical significance was denoted as *p < 0.05, **p < 0.01, and ***p < 0.001. Data are presented as mean ± SEM.

To further investigate the inflammatory response induced by the recombinant viruses, five cytokines (TNF-α, IL-1β, IL-5, IL-6, and IL-10) were measured in serum samples at 4 and 6 dpi following intranasal infection (**Figures 4A–E**). At 4 dpi, TNF-α, IL-5, and IL-10 showed no statistically significant differences among the three viral infection groups. Among all experimental groups, the Long-BAC group, used as the positive control, exhibited significantly higher expression levels of TNF-α (*p* < 0.05), IL-5 (*p* < 0.01), and IL-6 (*p* < 0.001) levels at 6 dpi than the other groups. Within the Long-BAC group, IL-1β (*p* < 0.05), IL-5 (*p* < 0.01), and IL-6 (*p* < 0.01) levels were significantly increased at 6 dpi compared with 4 dpi. IL-1β was elevated in the rLong- BJ1903-AF group at 4 dpi, whereas no significant between group differences in IL-1β or IL-10 were detected among the three viral groups. For the recombinant viruses, most cytokine levels at 6 dpi were numerically higher in rLong-SY2103-BF than in rLong-BJ1903-AF; however, these differences did not reach statistical significance.

## Discussion

Reverse genetics systems enable the systematic manipulation of viral genomes, facilitating the precise elucidation of how gene mutations modulate viral virulence (14–18). The F protein of HRSV is a critical determinant of viral pathogenesis and host immune responses (19–21). Globally, HRSV genotypes ON1 (HRSVA) and BA9 (HRSVB) are the most prevalent strains (10, 22, 23). Therefore, we developed a reverse genetics HRSV platform to explore how F protein genetic variations in these two dominant genotypes affect viral replication dynamics and pathogenicity.

In this study, we successfully generated two rRSVs, rLong-BJ1903-AF and rLong-SY2103-BF, by replacing the F gene within the Long-BAC isogenic backbone with F genes derived from distinct clinical isolates. Cellular-level characterization revealed altered replication kinetics for both recombinant strains compared with the parental Long-BAC virus. The recombinant strains exhibited reduced replication capacity, with peak viral genomic RNA loads delayed by ≥ 12 h. This phenotypic divergence strongly suggests a link to variations within the F protein. Sequence analysis revealed multiple amino acid differences in the F proteins of rLong-BJ1903-AF and rLong-SY2103-BF relative to the prototype Long strain, including 11 differences in BJ19–03 and 62 differences in SY21-03. These differences should be interpreted as sequence variations relative to the Long backbone and may include natural polymorphisms, subtype associated divergence, and strain specific differences rather than genotype-specific mutations alone. Notably, most of these substitutions were localized to the previously identified functional regions of the F protein. Previous studies have shown that residues within these regions can modulate key functional properties, including pre-F stability and membrane fusion activity. For instance, the K191R identified in rLong-SY2103-BF is localized within the HRA domain, a region involved in F-mediated membrane fusion, and residue 191 has been implicated, together with residue 79, in modulating the enhanced fusion phenotype of the HRSV line19 F protein (14). However, direct functional assays, such as fusion activity, pre-F stability, thermal stability, and site-directed mutagenesis analyses, were not performed in this study. Because each recombinant virus carried the complete F gene from a clinical isolate rather than individual engineered substitutions, the observed phenotypes cannot be attributed to any single amino acid change or defined combination of mutations. Future reverse-genetics studies involving site-directed mutagenesis and rescue of individual or combinatorial F substitutions in the same backbone will be required to identify the responsible residue(s).

Comparative analysis of the two recombinant strains revealed that rLong-BJ1903-AF reached higher peak viral genomic RNA levels than rLong-SY2103-BF under identical *in vitro* conditions (e.g., MOI, cell confluence, and incubation temperature). rLong-BJ1903-AF exhibited accelerated and more extensive syncytium formation. Together, these findings suggest that replication fitness is not determined simply by the number of F protein substitutions, but may depend on their location, combination, structural context, and compatibility with the Long-BAC backbone. The stronger *in vitro* growth of rLong-BJ1903-AF further suggests that the ON1 derived F protein may enhance replicative fitness within the Long-BAC genomic backbone, underscoring the potential functional significance of F protein sequence variation in membrane fusion (20, 24). However, because the multistep growth curves were based on viral genomic RNA quantification rather than infectious virus titration, differences in specific infectivity or non-infectious/defective particle production cannot be excluded. Future studies comparing PFU/genome copy ratios at matched time points will be needed to distinguish genome replication from infectious particle production.

To assess *in vivo* pathogenicity, BALB/c mice were intranasally inoculated with the recombinant viruses rLong-BJ1903-AF and rLong-SY2103-BF and the parental Long-BAC strain. The parental Long-BAC strain induced the most severe clinical manifestations, characterized by significant weight loss, elevated viral loads in the lung tissue, and exacerbated pulmonary pathology. rLong-SY2103-BF exhibited intermediate pathogenicity, whereas rLong-BJ1903-AF caused the mildest disease phenotype. However, because the maximum body weight loss induced by rLong-SY2103-BF was modest, body weight change alone may not fully reflect disease severity in this model and should be interpreted together with lung viral RNA burden and histopathological alterations. Notably, rLong-BJ1903-AF exhibited higher viral RNA levels and more extensive syncytium formation in HEp-2 cells but caused milder disease in mice, indicating that *in vitro* replication fitness does not necessarily predict *in vivo* virulence. This discordance may reflect differences in airway cell tropism, tissue spread, innate immune activation, epithelial damage, or host inflammatory responses. Since only the F gene was exchanged in the Long-BAC backbone, these data support an association between F protein sequence variation and pathogenic phenotypes within this experimental system. Nevertheless, the recombinant viruses do not fully recapitulate the genetic backgrounds of natural ON1 or BA9 viruses and were not directly compared with their corresponding parental clinical isolates, BJ19–03 and SY21-03. Therefore, extrapolation to naturally circulating strains should be made with caution, and future studies using additional contemporary clinical isolates or recombinant viruses with broader genomic backgrounds will be needed (11, 25).

To elucidate the inflammatory dynamics induced by recombinant viruses, serum cytokine profiles (TNF-α, IL-1β, IL-5, IL-6, and IL-10) were assessed at 4 and 6 dpi. At 4 dpi, TNF-α, IL-5, and IL-10 levels showed no significant differences among infection groups, whereas elevated IL-1β and IL-6 levels in the rLong-BJ1903-AF and Long-BAC groups suggested early proinflammatory responses that may be associated with differences in viral tropism or replication efficiency. At 6 dpi, the Long-BAC group showed significantly increased TNF-α, IL-5, and IL-6 levels, with no significant differences in IL-1β or IL-10 among the groups, indicating a sustained inflammatory response induced by this virus. Within the Long-BAC group, IL-1β, IL-5, and IL-6 levels were significantly higher at 6 dpi than at 4 dpi, further suggesting that Long-BAC may elicit a more persistent inflammatory response over time. In addition, most cytokine levels at 6 dpi, except IL-1β, tended to be higher in rLong-SY2103-BF than in rLong-BJ1903-AF, although these differences were not statistically significant, consistent with the more severe pathological changes observed *in vivo*. Together, these findings suggest that genotype-specific F protein variation may influence viral replication, tropism, and the magnitude and persistence of virus-induced inflammation, consistent with previous studies showing that the HRSV F protein can activate innate immune signalling pathways and promote cytokine production and epithelial damage (25, 26).

One limitation of this study is that the two recombinant viruses were administered at different infectious titers *in vivo* because of differences in rescue efficiency and virus stock titers. This may have affected direct quantitative comparisons of pathogenicity between the two recombinant strains. Nevertheless, rLong-SY2103-BF exhibited relatively greater pathogenicity despite the lower inoculation dose, including increased body weight loss, higher lung viral loads, and more severe histopathological changes. These findings suggest that the observed phenotypic differences are unlikely to be explained solely by inoculum variation and may instead reflect intrinsic genotype-associated viral properties. Future studies using normalized infectious doses will be necessary to enable more rigorous quantitative comparisons between recombinant strains.

This study provides evidence for potential links between F protein sequence variation in prevalent HRSV genotypes and key viral phenotypes, including replication efficiency, virulence, and host immune modulation. Notably, both recombinant viruses exhibited reduced replication capacity and less severe pathogenic phenotypes compared with the parental Long-BAC strain within this F gene replacement system. These findings provide a basis for future studies aimed at identifying virulence-associated residues in the F protein and informing antiviral and vaccine development. Systematic comparative analyses of the recombinant viruses, their parental clinical isolates BJ19–03 and SY21-03, and laboratory-adapted Long-derived strains in both cell culture and animal models will be important to distinguish F specific effects from whole-genome background effects.

## Data Availability

The original contributions presented in the study are included in the article/supplementary material. Further inquiries can be directed to the corresponding author.
